# How bacteria keep proteins moving

**DOI:** 10.7554/eLife.33590

**Published:** 2017-12-12

**Authors:** Conrad W Mullineaux

**Affiliations:** School of Biological and Chemical SciencesQueen Mary University of LondonLondonUnited Kingdom

**Keywords:** protein mobility, physicochemistry of cytoplasm, bacteria, intracellular diffusion, ionic strength, ribosomes, *E. coli*

## Abstract

Bacteria contain large numbers of negatively-charged proteins to avoid the electrostatic interactions with ribosomes that would dramatically reduce protein diffusion.

**Related research article** Schavemaker PE, Śmigiel WM, Poolman B. 2017. Ribosome surface properties may impose limits on the nature of the cytoplasmic proteome. *eLife* **6**:e30084. doi: 10.7554/eLife.30084

The cytoplasm of a bacterial cell is densely packed with DNA, RNA and various proteins and macromolecules (Zimmerman and Trach, 1991). In *Escherichia coli,* for example, the cytoplasm is home to around two million soluble protein molecules (estimated using data from the BioNumbers database) and about 55,000 ribosomes (Bakshi et al., 2012; see also Goodsell, 2009 for vivid illustrations of the dense packing of the bacterial cytoplasm). This overcrowding can have profound effects on the biochemistry of the cytoplasm and on its physical properties (Ellis, 2001; Parry et al., 2014). However, despite being congested with macromolecules, the cytoplasm remains surprisingly fluid.

For decades, researchers have used green fluorescent proteins, also known as GFPs, to study how molecules move in living organisms, because GFPs can be tracked with a fluorescence microscope. GFPs diffuse rapidly: for example, one GFP molecule can travel the entire length of an *E. coli* cell in less than one second (Nenninger et al., 2010). However, adding a small positively-charged ‘tag’ to the negatively charged GFP slows its diffusion in *E. coli* (Elowitz et al., 1999).

Nevertheless, it has remained unclear what factors control the diffusion of proteins in the cytoplasm. Now, in eLife, Paul Schavemaker, Wojciech Śmigiel and Bert Poolman of the University of Groningen report that the total surface charge of proteins strongly influences their mobility (Schavemaker et al., 2017). By systematically increasing the positive surface charge of GFPs in *E. coli* and two other prokaryotes, Schavemaker et al. showed that proteins with a positive charge moved up to 100 times more slowly than proteins with a negative charge.

Schavemaker et al. take the story further with elegant experiments that identify the main culprit: the ribosome. Ribosomes are bulky structures with a negative surface charge that mainly comes from their RNA (Knight et al., 2013). Moreover, they regularly interact with other macromolecules when they are translating mRNA transcripts to produce new proteins. Schavemaker et al. estimate that each ribosome is big enough to trap up to 66 positively-charged GFP-sized proteins on its surface. This can stop ribosomes from working, cause the cytoplasm to clot, and hinder any process that depends on the free movement of proteins.

So, how can this problem be avoided? A simple but drastic solution would be to ban positively-charged proteins from the cytoplasm (as the remaining negatively-charged proteins will not be attracted to the ribosomes in the first place). In fact, this seems to be the solution adopted by most prokaryotes. It has long been known that in most organisms, the majority of proteins in the cytoplasm have net negative charge at physiological pH (Schwartz et al., 2001).

Indeed, Schavemaker et al.’s detailed analysis of three different species of prokaryotes indicates that, with the exception of a few nucleic-acid binding proteins and ribosomal subunits, the cytoplasm is almost devoid of positively-charged proteins. This hints at a strong and previously unsuspected evolutionary pressure to ensure that proteins in the cytoplasm are negatively-charged.

However, not all prokaryotes follow this rule. Schavemaker et al. identify four bacteria that mainly have positively-charged proteins inside their cytoplasm. It is not known how these bacteria prevent the cytoplasm from clotting, but it could be significant that all four have small genomes and live in symbiosis with a eukaryotic host. The situation in eukaryotes also warrants a closer look: do our ribosomes influence the mobility of the proteins in our cytoplasm?

The findings of Schavemaker et al. have clear implications for biotechnologists who wish to engineer bacteria to produce foreign proteins, as it could be problematic to produce positively-charged proteins. Synthetic biologists working on the ambitious goal of turning bacteria into cell factories that produce entirely new products (Nielsen and Keasling, 2016) should also be wary of introducing positively-charged proteins into the cytoplasm.
